# Disruption of Broad Epigenetic Domains in PDAC Cells by HAT Inhibitors

**DOI:** 10.3390/epigenomes3020011

**Published:** 2019-06-02

**Authors:** Diana L. Gerrard, Joseph R. Boyd, Gary S. Stein, Victor X. Jin, Seth Frietze

**Affiliations:** 1 Biomedical and Health Sciences Department, University of Vermont, Burlington, VT 05405, USA;; 2 Cellular Molecular Biomedical Sciences Program, University of Vermont, Burlington, VT 05405, USA; 3 Biochemistry, University of Vermont, Burlington, VT 05405, USA;; 4 The University of Vermont Cancer Center, Burlington, VT 05405, USA; 5 Department of Molecular Medicine, University of Texas Health Science Center, San Antonio, TX 78229, USA;

**Keywords:** super-enhancers, epigenomics, pancreatic cancer

## Abstract

The spreading of epigenetic domains has emerged as a distinguishing epigenomic phenotype for diverse cell types. In particular, clusters of H3K27ac- and H3K4me3-marked elements, referred to as super-enhancers, and broad H3K4me3 domains, respectively, have been linked to cell identity and disease states. Here, we characterized the broad domains from different pancreatic ductal adenocarcinoma (PDAC) cell lines that represent distinct histological grades. Our integrative genomic analysis found that human derived cell line models for distinct PDAC grades exhibit characteristic broad epigenetic features associated with gene expression patterns that are predictive of patient prognosis and provide insight into pancreatic cancer cell identity. In particular, we find that genes marked by overlapping Low-Grade broad domains correspond to an epithelial phenotype and hold potential as markers for patient stratification. We further utilize ChIP-seq to compare the effects of histone acetyltransferase (HAT) inhibitors to detect global changes in histone acetylation and methylation levels. We found that HAT inhibitors impact certain broad domains of pancreatic cancer cells. Overall, our results reveal potential roles for broad domains in cells from distinct PDAC grades and demonstrate the plasticity of particular broad epigenomic domains to epigenetic inhibitors.

## 1. Introduction

Cancer is a complex disease arising from both genetic and epigenetic alterations that impact changes in gene expression to drive and maintain malignant phenotypes. In recent years, epigenomic profiling has revealed that cancer progression involves a global reprogramming of networks of functional DNA regulatory elements, including enhancers [1]. Enhancers are cis-acting elements that positively control the transcription of target genes and play central roles in regulating cell-type or tissue-type specific genes during development and differentiation [2]. Enhancer sequences are comprised of DNA sequence motifs that allow transcription factors to bind in a sequence-specific manner and to recruit various histone writers to regulate transcriptional regulation. Recently, clusters of enhancer elements, referred to as super-enhancers, have been linked to cell identity and disease states [3–7]. In addition, regions with widespread H3K4me3 modification, called broad H3K4me3 domains, have also emerged as important domains linked to the expression of tumor suppressor and cell identity genes [8,9]. Understanding the functional roles of these epigenomic domains in different cancer types has the potential to uncover new strategies for the development of new cancer therapies [10].

Pancreatic ductal adenocarcinoma (PDAC) is the most common form of pancreatic cancer and ranks as one of the deadliest diseases with a relative five-year survival rate of approximately 9% [11]. PDAC is associated with several genetic and epigenetic alterations, leading to the activation of growth promoting and cell survival pathways and the inactivation of apoptotic and tumor suppressor pathways [12]. Recent reports have demonstrated the PDAC enhancer landscape and have classified enhancers associated with PDAC progression [13–15]. Additionally, the super-enhancer landscape has also been investigated in the squamous PDAC subtype [16–18]; however, the broad H3K4me3 landscape has largely been unexplored in this cancer.

To increase our understanding of broad epigenomic domains and their association with PDAC gene regulation in cancer progression, we classified super-enhancer and broad H3K4me3 domains in widely studied human PDAC cell lines. We specifically defined groups of epigenomic domains that correspond to distinct histological grades and compared their enriched pathways and associated gene expression levels within these domains. We show that broad domains correlate with clinical features and hold potential as markers for patient stratification. As epigenetic inhibitors are promising avenues for cancer treatment, we also explored the genome-wide broad domain targets of two histone acetyltransferase (HAT) inhibitors.

## 2. Results

### 2.1. Classifying the Broad Domains of Different PDAC Cell Lines

**Figure 1.**
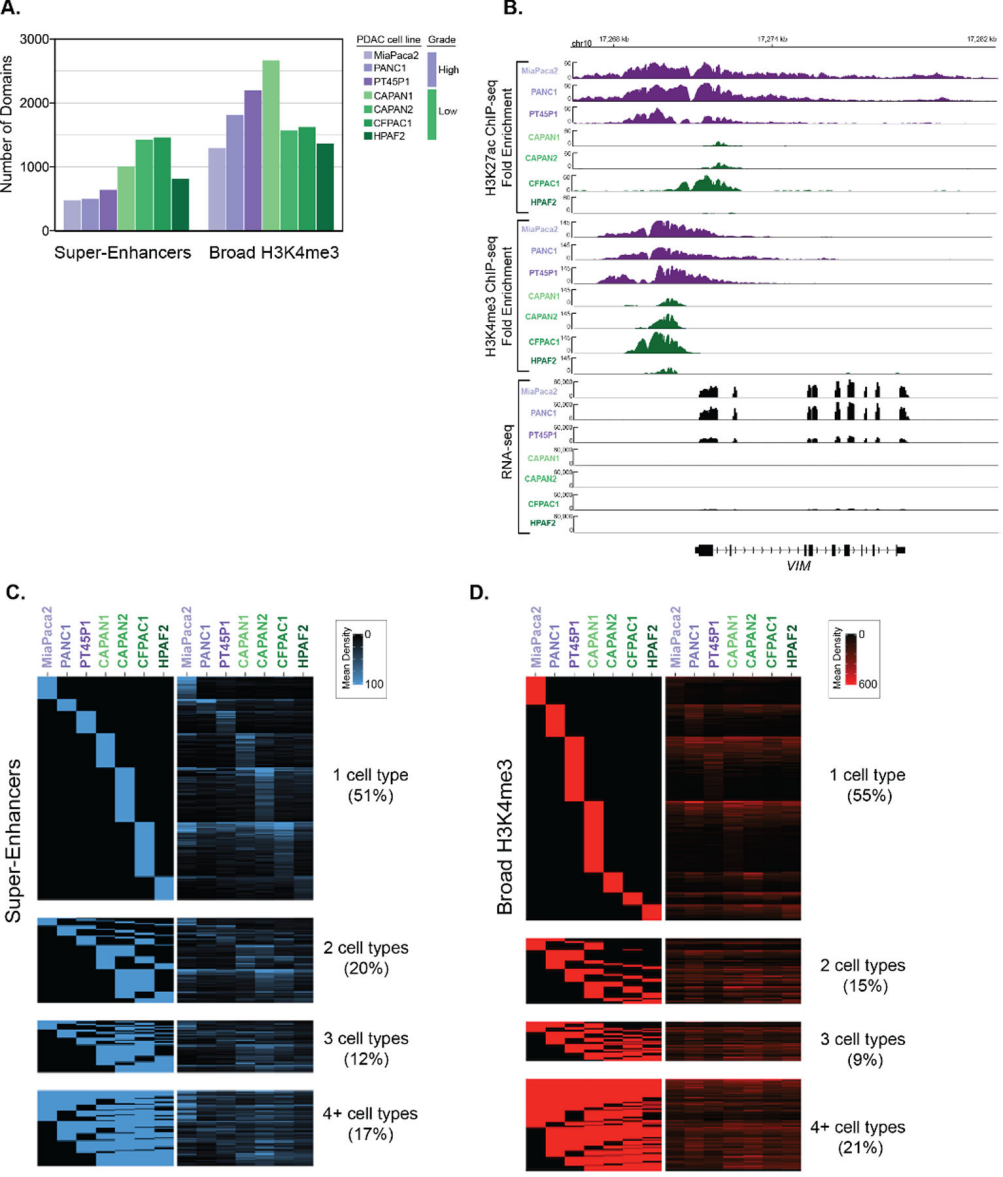
Determination of super-enhancers and broad H3K4me3 domains in PDAC cell lines that correspond to different histological grades. **(A)** The total number of super-enhancers and broad H3K4me3 domains found in different human PDAC cell lines that represent either High-Grade (purple) or Low-Grade (green) PDAC groups. **(B)** Genome browser representation of the H3K27ac and H3K4me3 ChIP-seq signal, as well as the RNA-seq signal over an approximate 14 kb region surrounding the *VIM* locus. **(C,D)** Heatmaps displaying the classification of super-enhancer **(C)** and Broad H3K4me3 **(D)** domains across seven human PDAC cell lines. The rows represent individual regions and the columns represent the different cell lines. The left panel heatmap is a binary heatmap displaying a region overlap between the cell lines, and the right panel is a signal heatmap of H3K27ac or H3K4me3 signal for **(C)** and **(D)** respectively. The color scale represents the signal density of either H3K27ac or H3K4me3, respectively.

We analyzed ChIP-seq data from different human PDAC cell lines to identify super-enhancer and broad H3K4me3 domains, respectively [13]. This data was derived from a panel of human PDAC cell lines that are representative of both Low and High PDAC tumor grades, based on genotypic and phenotypic characteristics [19–21]. For example, the ‘High-Grade’ PANC1, MiaPaCa2 and PT45P1 cell lines all express mesenchymal genes [13,21], show mesenchymal spindle-shaped cell morphology [22], and are considered to be poorly differentiated [19,23,24]. In contrast, the ‘Low-Grade’ PDAC cell lines CAPAN1, CAPAN2, CFPAC1 and HFPAC1 display epithelial-like features and are considered to be well-differentiated [25–28]. To determine the super-enhancer domains using these different PDAC datasets, we essentially followed the same procedures used in Hnisz et al. [3] with some slight modifications. Briefly, H3K27ac peaks were called from ChIP-seq data against input and enriched peaks that clustered within 5 kb were stitched together. These stitched regions were then ranked to determine super-enhancers. Broad H3K4me3 domains were identified from enriched peaks identified from ChIP-seq, where the top 5% of peaks based on domain size were used to call broad H3K4me3 domains [29]. In total, we identified between 457 and 1346 super-enhancers and 1214 to 2559 broad H3K4me3 domains in seven different PDAC cell lines that correspond to Low- and High-Grade groups, respectively (Figure 1A). We observed that many genes were differentially marked by broad domains, according to the assigned PDAC Grade group. As an example, the *VIM* gene encoding VIMENTIN, which is central to metastasis, is bound by both types of broad epigenetic domains in all High-Grade cell lines and corresponds with high levels of gene expression in these cells (Figure 1B). We inspected the profiles of other regulatory histone modifications within the broad epigenetic domains using ENCODE data from PANC1 cells [30]. The PANC1 super-enhancers defined by ranked ordering exhibit higher H3K4me1 signal compared to typical enhancers for both distal and proximal regions (Figure S1A,B). The H3K4me3 signal was enriched in proximal typical and super-enhancers compared to their distal counterparts, as well as both similar and distinctive gene ontology categories (Figure S1C). Both typical and broad H3K4me3 domains display low H3K4me1 enrichment, whereas broad H3K4me3 domains display higher H3K27ac than typical H3K4me3 regions (Figure S2). Comparison of the ChIP-seq signal of the different domains between cell lines reveals that the majority of super-enhancers, and broad H3K4me3 domains are uniquely enriched in a given cell line (Figure 1C,D).

**Figure 2.**
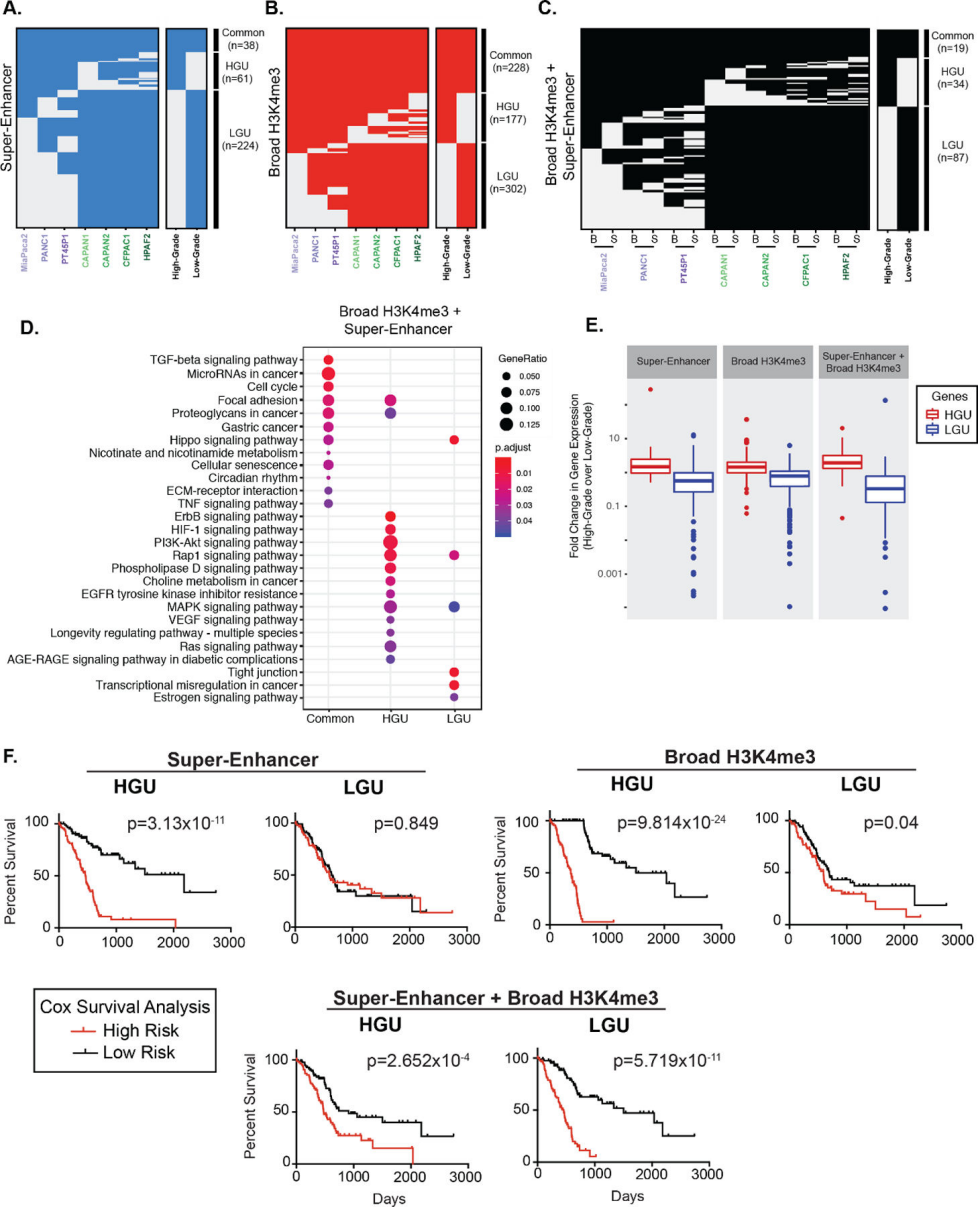
Broad domains mark distinctive pathways and are predictive of poorer PDAC patient survival. **(A)** Clustering of genomic regions encompassing super-enhancers across seven human PDAC cell lines to define common, High- and Low-Grade unique super-enhancers. **(B)** A similar analysis was performed on broad H3K4me3 domains to define common, High- and Low-Grade unique (HGU and LGU, respectively) broad H3K4me3 domains. **(C)** A similar analysis was performed to look at overlapping domains, where ‘B’ represents Broad H3K4me3 domains and ‘S’ represents super-enhancers. **(D)** Gene Ontology pathway enrichment profiles of genes marked by both domains that are common, HGU or LGU domains. **(E)** Comparison of expression levels of genes marked by the indicated domains in High-Grade and Low-Grade PDAC cells. Data is derived from mean normalized expression counts of genes. **(F)** Kaplan-Meier survival analysis of high- and low-risk groups (red and black, respectively) for genes marked High-Grade unique (HGU) and Low-Grade unique (LGU) domains for the indicated domain type.

We hypothesized that cells in separate differentiation states exhibit characteristic broad epigenetic patterns. We, therefore, compared the regions between different PDAC cell lines to define Grade-spacific broad domains. Altogether, 38 super-enhancers were common to all PDAC cell lines, and 61 and 224 super-enhancers were unique to High-Grade and unique to Low-Grade groups (HGU and LGU, respectively) (Figure 2A). Similarly, we identified 228 common and 177 HGU and 302 LGU broad H3K4me3 domains (Figure 2B). We further compared the overlap of both types of broad domains for the different PDAC groups by clustering these regions. There were 87 overlapping super-enhancer and broad H3K4me3 domains for LGU, compared to the 34 overlapping HGU domains (Figure 2C). In general, Low-Grade PDAC cells had an increased number of super-enhancer domains compared to High-Grade PDAC cells, whereas both groups have a similar number of broad H3K4me3 domains. Overall, this analysis revealed that distinct human derived PDAC cell line models corresponding to PDA3 Grades exhibit characteristically broad epigenomic domains.

### 2.2. Broad Epigenomic Domains Mark Distinct PDAC Pathways

Prior studies have demonstrated that broad domains are associated with developmental and cell identity genes and broad H3K4me3 domains, in particular, have been shown to mark tumor suppressor genes [8,9,31]. To explore the gene pathways associated with super-enhancers and broad H3K4me3 domains, we annotated genes marked by each type of domain and determined their functional classifications (Figure S3and Figure 2D). Interestingly, pathway enrichment analysis showed that the genes marked by overlapping domains are involved with a variety of signaling pathways that were either specific to LGU, HGU, or common to all PDAC cells. For example, pathways that enriched common to all PDAC cells included transforming growth factor beta (TGFβ), microRNAs in cancer and cell cycle. Pathways specific to HGU included vascular endothelial growth factor (VEGF) and retrovirus-associated DNA sequences (Ras) signaling pathways, whereas pathways specific to LGU had a tight junction and Hippo signaling (Figure 2D).

### 2.3. Broad Regions Are Associated with Poorer Survival in PDAC Patients

Super-enhancers and broad H3K4me3 domains have been linked to increased gene expression in a variety of tissue types [32,33]. We, therefore, inspected the relative expression levels of genes marked by the different broad domains across PDAC Grades using available RNA-seq data from the corresponding cell lines [13]. As expected, both super-enhancer and broad H3K4me3 domains unique to each Grade group (LGU and HGU) showed appreciably elevated expression in the corresponding group compared to the contrasting group (Figure 2E and Figure S4). For example, genes marked by HGU super-enhancers had significantly higher expression levels in High-Grade cells compared to Low-Grade cells.

We next explored the clinical association of the gene expression for genes uniquely marked by different broad domains relative to patients’ overall survival using the TCGA PDAC dataset [34]. Kaplan-Meier survival analysis showed that gene expression marked by HGU super-enhancers, as well as HGU broad H3K4me3 domains, are strong predictors of poor survival (Figure 2F). In contrast, gene expression linked to LGU domains do not predict a poorer survival rate. However, the expression levels of genes bound by both LGU super-enhancers and broad H3K4me3 domains are significantly associated with a worse overall survival in PDAC patients (Figure 2F). Overall, these results reveal that genes uniquely bound by different domains are predictive of PDAC patient outcome.

### 2.4. Histone Acetyltransferase Inhibitors Alter Global H3K27ac and H3K4me3 Levels

**Figure 3.**
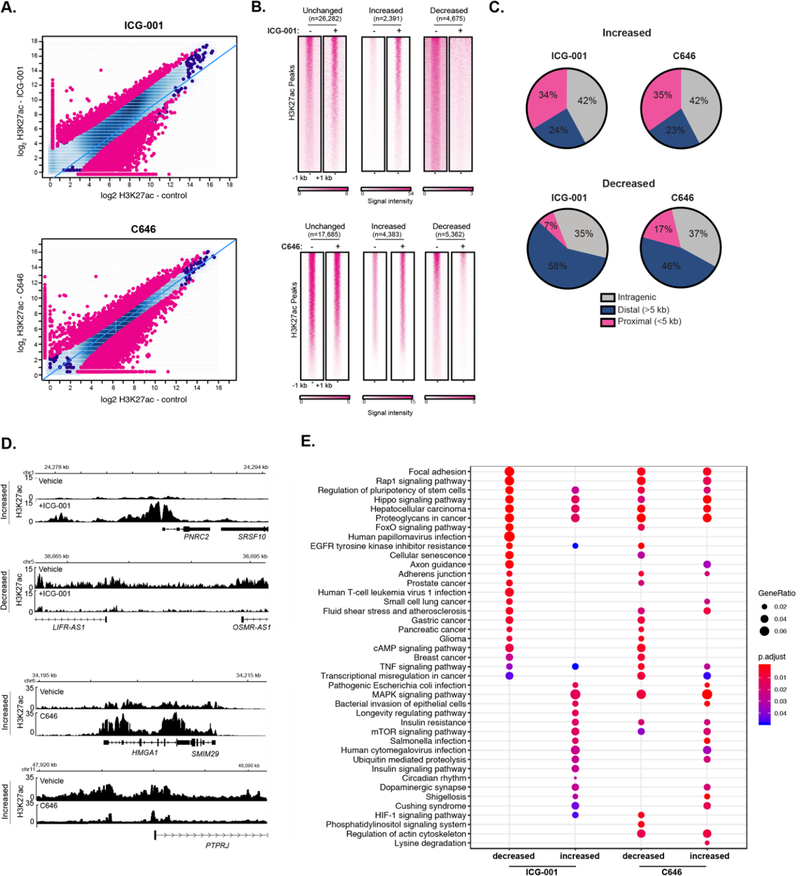
Inhibitors of histone acetyltransferases impact global H3K27ac levels. **(A)** Differential H3K27ac enrichment analysis reveals significantly altered genome-wide H3K27ac sites in response to ICG-001 **(Top)** or C646 **(bottom)** treatment (FDR < 0.1), signal is represented as log_2_ normalized read count for the indicated condition. **(B)** Signal heatmaps representing the H3K27ac within altered regions after ICG-001 or C646 treatment identified from the differential analysis. **(C)** Location analysis of increased or decreased H3K27ac signal after treatment relative to gene regions. **(D)** Example of genes with increased or decreased H3K27ac signal after ICG-001 treatment; the signal is represented as fold enrichment over input. **(E)** KEGG pathway enrichment analysis of genes within altered regions after HAT inhibitor treatment.

Epigenetic modulation via small molecule inhibitors has been proposed as an approach for treating various malignancies, including pancreatic cancer [35,36]. We have previously shown that the two HAT inhibitors, ICG-001 and C646, differentially impair the global gene expression levels in human pancreatic and colorectal cancer cell lines [37]. Additionally, other work has shown that ICG-001 and C646 reduce tumorigenicity of pancreatic cancer cell models [38–40] and ICG-001 has been used in clinical trials for pancreatic cancer (). However, the impact of HAT inhibitor treatment on histone acetylation remains unknown. To determine the effect of HAT inhibitors on genome-wide H3K27ac enrichment, we treated PANC1 cells with ICG-001, C646, or a vehicle control and performed H3K27ac ChIP-seq, each with biological replicates. As expected, HAT inhibitor treatment caused significant changes in genome-wide H3K27ac patterns compared to vehicle treatment (Figure 3A). Differential H3K27ac analysis showed 4675 and 5362 regions with reduced H3K27ac levels in ICG-001 and C646 treated cells, respectively (false discovery rate (FDR) < 0.1). There were also 2391 and 4383 sites with elevated H3K27ac levels in ICG-001 or C646 treated cells, respectively (Figure 3B). Interestingly, for either treatment, the majority of higher H3K27ac enrichment clustered within the gene body (intragenic regions), whereas the bulk of reduced H3K27ac enrichment corresponded to distal intergenic regions (Figure 3C). Examples of genes that display altered H3K27ac patterns for each treatment are shown in Figure 3D. Pathway enrichment analysis indicated that HAT inhibitor treatments influence H3K27ac enrichment at genes that map to a variety of pathways (Figure 3E). For example, both treatments decreased H3K27ac levels at genes that belong to pancreatic, glioma, breast, and gastric cancers, whereas C646 decreased the H3K27ac at HIF-1 and phosphatidylinositol signaling gene pathways.

We further investigated the impact of ICG-001 treatment on global H3K4me3 levels in PANC1 cells. ChIP-seq for H3K4me3 showed global H3K4me3 enrichment alterations in ICG-001 treated PANC1 cells. In total, there were 6847 increased and 3219 decreased regions (Figure S5A). *PPP2R2C*, a tumor suppressor gene [41], exhibited elevated H3K4me3 signals with ICG-001 treatment compared to the control. In contrast, *NKIRAS1*, encoding a RAS-like protein, exhibited a decreased H3K4me3 signal with ICG-001 treatment (Figure S5B).

### 2.5. HAT Inhibitors Alter PDAC Broad Epigenomic Domains

**Figure 4.**
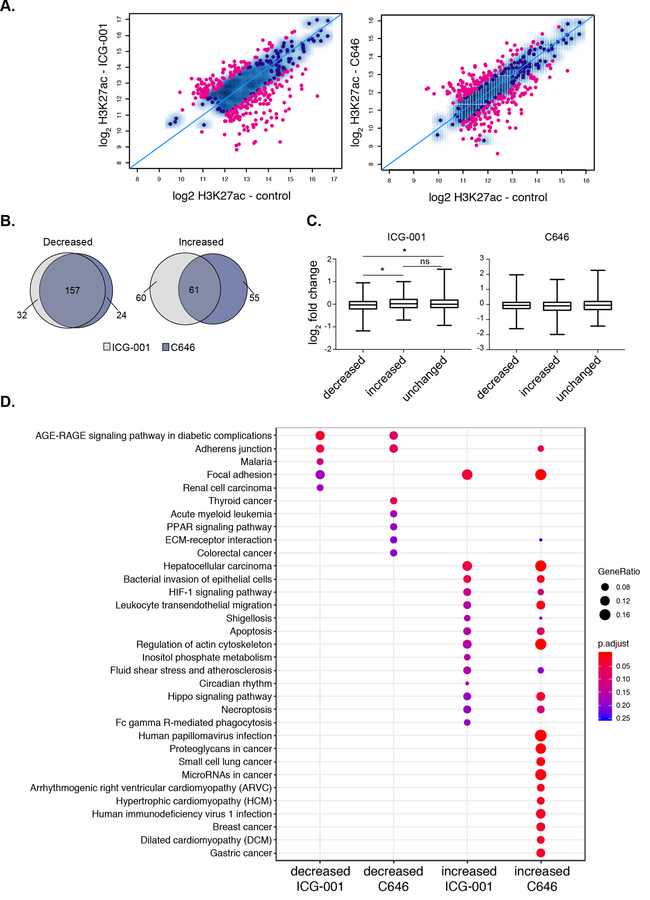
Histone acetyltransferase (HAT) inhibitors influence the acetylation levels at super-enhancers. **(A)** Scatterplot displaying the differential enrichment of H3K27ac (log2 ChIP-seq read count) for ICG-001 **(left)** and C646 **(right)** compared to control; the blue diagonal line separates those of increasing or decreasing signals with the colored dots corresponding to regions with significant changes in treatment compared to control (FDR < 0.1). **(B)** Overlap analysis of regions comparing the increased or decreased H3K27ac regions after ICG-001 and C646 treatment. **(C)** Boxplots displaying log2 fold change of genes (treatment vs. control) within the given differential domains identified in **(A)** (Welch’s *t*-test, * represents *p* < 0.05). **(D)** Pathway enrichment analysis of genes within altered super-enhancers after ICG-001 or C646 treatment.

We next determined the impact of HAT inhibitor treatment on H3K27ac signal at super-enhancers and found that both treatments specifically impact H3K27ac enrichment levels at many super-enhancers (Figure 4A). In total, there were 136 and 128 super-enhancers with reduced H3K27ac levels, whereas 121 and 117 super-enhancers showed significant gains in H3K27ac levels after ICG-001 and C646 treatments, respectively (FDR < 0.1). Notably, most super-enhancers with decreased H3K27ac are the same between either treatment (~90%), whereas only ~50% of the domains with increased H3K27ac are the same between either treatment (Figure 4B). We next used available microarray gene expression data from PANC1 cells with identical treatments [37] to determine the impact of inhibitors on the expression of genes that are associated with altered H3K27ac levels. Increases in H3K27ac signal at super-enhancers was associated with elevated gene expression levels and similarly decreased enrichment at super-enhancers corresponded to a reduced gene expression levels, but only with ICG-001 treatment (Figure 4C). Pathway enrichment analysis showed that different pathways are linked to the super-enhancers targeted by HAT inhibitors (Figure 4D). ICG-001 treatment targets super-enhancers that correspond to genes involved with AGE-RAGE signaling complications in diabetes. In addition to super-enhancers, ICG-001 treatment resulted in 4 decreased and 113 increased broad H3K4me3 domains. A comparison of both domains targeted by ICG-001 treatment reveals an increase of H3K27ac and H3K4me3 enrichment at 24 distinct genomic regions (Figure S5). Overall, these results delineate the broad epigenomic domains that are sensitive to HAT inhibitor treatment in PANC1 cells.

### 2.6. HAT Inhibitor Treatment Targets Broad Domains that Are Enriched at TAD Boundary Regions

**Figure 5.**
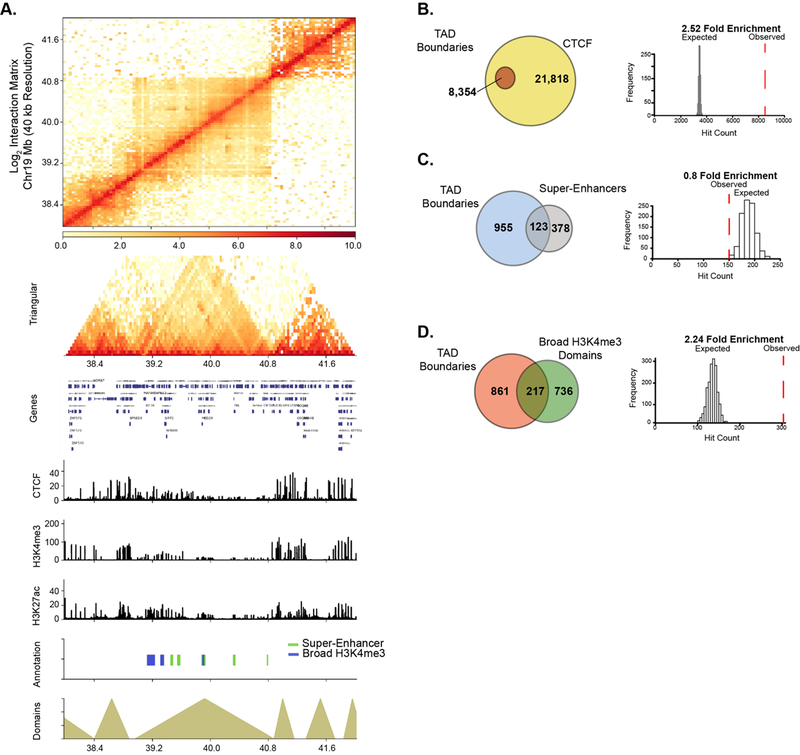
Broad domains are linked to topological associated domain boundaries. **(A)** Chromatin interaction matrix at 40 kb resolution showing broad domains contained within topologically associated domain (TAD) regions. Statistical associations of TAD boundaries were performed for **(B)** CTCF, **(C)** super-enhancers and **(D)** Broad H3K4me3 domains.

Recently, super-enhancer domains that overlap with broad H3K4me3 domains were shown to be linked to higher-order chromatin interactions, signifying a unique spatial organization of chromatin around cell-specific epigenetic domains [8,42], We, therefore, examined the relationship between higher-order chromatin organization and broad epigenetic domains in PANC1 cells. Genome-wide chromatin contacts were determined by analyzing tethered-chromatin conformation capture (TCC) data in PANC1 [43]. Chromatin contacts were partitioned into topologically associated domains (TADs) and TAD boundary regions using a resolution of 40 kb (Figure 5A and Figure S6). We examined TADs in relation to CCCTC-binding factor (CTCF) and PANC1 broad domains. As expected, CTCF was significantly enriched at TAD boundaries (Figure 5B). Similarly broad H3K4me3 domains were significantly linked to TAD boundaries, however super-enhancers were not found to be enriched at TAD boundaries (Figure 5C,D). To study if HAT inhibitor-sensitive domains are linked to higher-order chromatin structures, we investigated the significance of association of both types of broad domains that are impacted by ICG-001 treatment with TAD boundaries. We found that the domains with increased H3K4me3 and H3K27ac enrichment were significantly associated with TAD boundaries, whereas domains with decreased enrichment were not associated with TAD boundaries (Figure S7). Thus, these results show that broad domains that gain enrichment of either active histone modification are linked to TAD boundaries. Overall, this analysis demonstrates the global impact of drug treatment on the epigenome and shows that certain classes of broad domains within TAD boundaries are sensitive to epigenetic inhibitors.

## 3. Discussion

The extension of epigenetic regulatory domains has emerged as a diagnostic marker that can serve to distinguish cancer cell identity and disease states. Accordingly we characterized the broad domains in several different cell lines that represent distinct PDAC histological grades. Previous work has investigated the factor-mediated enhancer reprogramming of the aggressive squamous PDAC subtype and identified a relationship between super-enhancer regions and known oncogenic factors [16,17]. Another study found similar regions to be regulated by the histone demethylase KDM6A with loss of KDM6A resulting in sensitization of pancreatic cancer cells to bromodomain inhibitors [18]. Moreover, recent work characterized two classifications of pancreatic cancer subtypes and identified their epigenomic landscapes, including super-enhancers in patient derived tumor xenografts [44], While these studies have provided extremely valuable insight into factor mediated regulation of super-enhancers, super-enhancers have not been globally characterized in an integrative analysis of widely used High- and Low-Grade PDAC cell lines. By clustering the domains from seven different PDAC cell lines into High- and Low-Grade groups, we find that different human derived PDAC cell line models corresponding to PDAC grades exhibit characteristic epigenetic features that identify gene expression patterns that are predictive of PDAC patient prognosis and provide insight into pancreatic cancer cell identity. Of particular interest are the genes marked by both super-enhancer and broad H3K4me3 domains in Low-Grade groups. Low-Grade groups demonstrate an enrichment for a greater number of unique gene pathways, which include several pathways significant to PDAC progression. Such pathways include tight junction, glycerophospholipid and Rap1 signaling pathways. We also provide evidence that genes marked by overlapping Low-Grade broad domains correspond to epithelial phenotype and hold potential as a marker for patient stratification (Figure 2). Thus, different PDAC grades exhibit characteristic pathways marked by broad epigenomic domains that provide insight into pancreatic cancer cell identity in the context of PDAC progression.

PDAC broad domains span numerous distinctive loci including the homeobox (*HOX*), small mothers against decapentaplegic (*SMAD*), and forkhead box *(FOX)* family of genes, proteins that have known roles in cell-type specific functions and are known factors in PDAC tumor cell biology [45–47], After annotating the genes marked by different broad domains, we identified known PDAC signaling pathways including the TGFβ and mitogen-activated protein kinase (MAPK) pathways, which are downstream effectors of oncogenic Kirsten rat sarcoma viral oncogene homolog (KRAS). Oncogenic KRAS is an established driver of pancreatic cancer and several pathways that are known downstream effectors of KRAS signaling and play central roles in PDAC cancer cell growth and survival [48–50], were found to be marked by broad domains in domains common to all PDAC cells. Super-enhancers common to all PDAC cell lines are significantly enriched with a variety of cancer signaling pathways, including focal adhesion, PI3K-AKT, microRNAs, and Hippo signaling. LGU super-enhancers were uniquely associated with several pathways that include tight junction, Rap1 signaling, and glycerophospholipid metabolism. Aberrant lipid synthesis and the reprogramming of lipid metabolism has been associated with the development and progression of pancreatic cancer [51], and several phospholipids have been identified as potential biomarkers in different types of pancreatic cancers [52,53]. MAPK signaling was the singular KEGG pathway enriched in HGU super-enhancers (Figure S3). Similarly, the broad H3K4me3 domains common to all PDAC groups were associated with distinctive pathways, including transcription corepressor, protein kinase, cadherin binding, and RNA binding pathways (Figure S3). Several enriched pathways linked to LGU broad H3K4me3 domains include SMAD, protein kinase C, TGFβ, and β-catenin pathways, whereas the HGU broad H3K4me3 domains solely mark genes enriched in transcriptional corepressor pathways. Examples of broad PDAC epigenomic domains encompassing disease-associated genes include *SMADs* and forkhead box C2 (*FOXC2*) for super-enhancer regions and avian myelocytomatosis viral oncogene homolog (*MYC*) and cyclin D1 (*CCND1*) for broad H3K4me3 domains (Figures S1 and S2).

Interestingly, our analysis indicates that Low-Grade unique broad H3K4me3 domains are enriched for TGFβ signaling pathways. TGFβ acts as a tumor suppressor with growth-inhibitory activity in epithelial cells during early pancreatic tumorigenesis. However, TGFβ appears to promote tumor progression in advanced disease [54], We also found that broad epigenomic domains mark several other pathways with less well-characterized roles in PDAC tumor biology, including microRNAs and proteoglycans in cancer.

H3K4me3 has been widely recognized as a mark of active promoter regions [55]. Recent studies have correlated broad H3K4me3 domains with enhancer activity at tumor suppressor genes in normal and cancer cells to provide mutation-independent insight into tumor suppressor pathways of disease states [9]. Here, we found that broad H3K4me3 domains span a number of genes including *HOX*, MYC and *CCND1* genes. As super-enhancers and broad H3K4me3 domains have been shown to function coordinately through chromatin interactions [8,42], we identified regions containing both domains in both High- and Low-Grade cells. As mentioned previously, the expression of genes marked by both domains is significantly associated with poor prognosis in pancreatic cancer patients.

An improved understanding of PDAC tumor biology and tumor grading should leverage available therapies and data. While drugs that target epigenetic mediators are currently in development [56, 57], the mode of action of existing drugs has not yet been thoroughly examined. In particular, epigenome-wide studies of their effects remain largely undetermined. In this study, we utilize ChIP-seq to compare the effects of treatment with C646, which is a competitive inhibitor of both p300 and CREB binding protein (CBP) [58] to the effects of ICG-001, which prevents CBP interaction with the co-activator, p-catenin [59,60]. Indeed, following treatment with either drug, we detect global changes in histone acetylation levels. Our results suggest that, in general, these two drugs have similar effects on the epigenome of PDAC cells; however, we were able to identify drug-specific responses after treatment.

In our previous study, we investigated the impact of ICG-001 and C646 on the cancer transcriptome of colorectal and pancreatic cancer cell lines [37], Though these drugs have been appreciated to be inhibitors of the Wnt/β-catenin pathway [59,60], surprisingly in our transcriptome study we did not observe this pathway as a top pathway altered in the pancreatic cancer cell model. In this present study, we sought to expand our investigation by determining how these inhibitors specifically impact the active broad epigenomic landscape of a High-Grade pancreatic cancer cell model to deepen our understanding of other potential epigenomic and gene targets under control of these inhibitors. Additionally, ICG-001 has been used in clinical trials for pancreatic cancer (); however, its influence on the whole epigenome is largely unknown. We observed dramatic effects on the epigenome upon treatment with either ICG-001 or C646, with hundreds of regions showing differential enrichment of H3K27ac or H3K4me3. Interestingly, both drugs targeted similar super-enhancers, causing a reduction in histone acetylation levels near genes involved in pancreatic cancer and other solid cancers (Figure 4). Since it is of current interest to target super-enhancers, we find that both super-enhancers and broad H3K4me3 domains are sensitive to epigenetic modulation. Interestingly, we find enhanced enrichment of H3K27ac at specific super-enhancers with HAT inhibitor treatment. This result illustrates the complexity of epigenomic control and could be due to altered regulation of transcriptional complexes containing HDAC enzymes that remove histone acetylation. It is further possible that nucleosomes are altered in these specific regions. Our studies only investigate the specific patterns of H3K27ac or H3K4me3 modifications and do not include a total H3 analysis. Future work can be performed to better understand the nature of these alterations. Thus, our results provide insight into the plasticity of these domains in response to epigenetic modulation. Future work will be able to tailor these therapeutics to target such domains involved with specific cellular pathways involved with PDAC tumorigenesis.

In summary, our data provide new perspectives on the effect of HAT inhibitors on the epigenome and provide knowledge of the broad domains unique to different histological grades of human pancreatic cancer cell line models. Our data show that epigenomic domains that correlate with clinical features are plastic and hold potential as markers for patient stratification.

## 4. Materials and Methods

### 4.1. Cell Culture and Epigenetic Inhibitor Experiments

The human cell line PANC1 (ATCC #CRL-1469) was obtained from the American Type Culture Collection. The cells were cultured in Dulbecco’s modified Eagle’s medium supplemented with 10% fetal bovine serum and 1% penicillin/streptomycin. We obtained ICG-001 from Michael Kahn (University of Southern California; Los Angeles, CA, USA) and C646 from VWR (catalog# 102516–240, Franklin, MA, USA). Cells were grown to 70% confluency followed by treatment with 10 μM ICG-001 or 10 μM C646 and were collected after 12 h.

### 4.2. ChIP-Sequencing

After a 12-h incubation with either ICG-001 or C646, cells were crosslinked with 1% formaldehyde (Thermo Scientific #28908, Waltham, MA, USA) for 10 min and quenched with 0.125 M Glycine. The ChIP-seq experiments were further performed as described by O’Geen et al. [61] and the antibodies used for the given targets were as follows: H3K27ac (Abeam, Cambridge, MA, USA; Ab4729 lot#GR16377-l) and H3K4me3 (Abeam; Ab8580). We performed duplicate ChIP-seq experiments for each histone. For each histone ChIP-seq assay, 10 μg of chromatin was incubated with (2.5–5 μg) of antibody. To confirm enrichment of target sequences, we performed qPCR in ChIP versus input samples. DNA was quantified using Qubit (Invitrogen, Waltham, MA, USA) and libraries were prepared using the NEBNext ChIP-seq Illumina Sequencing library preparation kit with 12 cycles of PCR for each library (New England Biolabs, Ipswich, MA, USA). Libraries were pooled and sequenced on the Illumina (San Diego, CA, USA) HiSeq2000 using single end 50 bp sequencing.

### 4.3. Tethered Chromatin Capture (TCC)

TCC was performed as detailed by Kalhor et al. [62], Briefly, approximately 5 × 10^7^ PANC1 cells were crosslinked as described above for ChIP-seq experiments and cell pellets were collected and stored at −80 °C until TCC experiment was carried out. Nuclei were digested with 2000 U H indIII (NEB, Ipswich, MA, USA) prior to dilute solid-surface ligation reactions and TCC library preparation was performed as described [62], The TCC dataset is available through GEO with the accession GSM1684570.

### 4.4. ChIP-Sequencing Analysis

ChIP-seq datasets were obtained from GSE31755, GSE64557, and GSE68858. For all datasets, raw sequencing reads were aligned to the human reference genome hg19 using bowtie2 with default parameters (Table S1) [63]. We determined the binding sites of each ChIP-seq experiment using model-based analysis of ChIP-seq (MACS2) with default parameters, with the exception of using the flag ‘-broad’ for determining broad H3K4me3 binding sites [64], Super-enhancer regions were identified over typical-enhancer regions using the Ranked Ordering of Super-enhancer (ROSE) tool [5,65]. Briefly, peaks were called from the H3K27ac ChIP-seq data and stitched together in 12.5 kb windows, which were further used to identify super-enhancers (ranked cutoff score of 19,701.68). Broad H3K4me3 regions were obtained via filtering for the top 5% of peaks (largest by domain size). Enrichment of signal within regions was plotted with the functions ‘plotProfile’ and ‘plotHeatmap’ within deepTools [66]. Overlapping binding regions were determined using peak interesectR. To determine peak locations relative to gene regions, we utilized the ‘annotatePeaks.p1’ function within HOMER [67], Visualization snapshots of ChIP-seq regions were obtained by building a signal track via the ‘bdgcompare’ utility in MACS [64], Bigwigs were obtained by ‘bedGraphToBigWig’ via UCSC tools [68] and further visualized using Integrated Genomic Viewer [69,70].

### 4.5. Coordination of Grade-Specific Broad Domains

H3K4me3, H3K27ac, and control ChIP-seq datasets for PDAC cell lines were obtained from GSE64557 and processed as described above. We utilized the Bioconductor package ‘seqsetvis’ (version 1.2.0, Burlington, VT, USA) [71] to visualize the distribution and overlap of genomic regions. The heatmaps comparing broad domains between different cell lines (Figure 1C,D) were generated in the following manner. First, overlapping regions were determined and plotted as a binary heatmap (left panel) to isolate regions that are unique to a given cell line, common in two, common in three, or shared in 4 or more cell lines. We then demonstrated the histone modification signal for these marks within the broad regions determined to display the strength of the histone modification signal within these regions and display this in the heatmap on the right.

### 4.6. Differential Binding Analysis ofChIP-Seq Datasets

To determine differential typical and broad H3K27ac and H3K4me3 regions, we used the DiffBind R package (version 2.10.0, Cambridge, UK) [72,73] with an FDR cutoff of <0.1.

### 4.7. Pathway Enrichment Analysis

Annotation of enriched gene pathways was performed using either ClusterProfiler (version 3.12.0, Guangzhou, China) or the Stanford GREAT tool (version 3.0.0, Stanford, CA, USA) [74,75].

### 4.8. Integration of Gene Expression Datasets

For integrating High- and Low-Grade gene expression for the different cell lines, we retrieved the corresponding RNA-sequencing expression datasets (GSE64558). We mapped these datasets to the human genome reference, hg19 and gene counts were normalized using DESeq2 (version 1.22.1) [76]. The mean of the normalized counts between replicates of each cell line was used to generate expression heatmaps. ICG-001 and C646 Illumina beadchip expression datasets were obtained from our previous study (GSE64038). We overlapped these differentially expressed genes within treatments with our genes annotated within drug altered broad regions to demonstrate expression changes at these genes. To assign genes within broad domains, we annotated within 100 kb of these regions using (GREAT) [68,69]. Any of the genes assigned within a given broad domain were coordinated with the gene expression datasets.

### 4.9. Topological Domains from TCC Data

Paired raw reads of the TCC data for the PANC1 cell line was processed using the HiC-Pro pipeline (version 2.11.0, Paris, France) [77]. Briefly, these reads were aligned to the human reference genome hg19 using bowtie2 with default parameters [63]. Then, 25 bp of the reads were trimmed and the reads were aligned iteratively. Reads with a MAPQ score less than 30 were removed, and the fragments were filtered for self-ligated fragments, duplicated reads from PCR, and error-pairs. Domains were detected using TopDom (version 0.0.2, Los Angeles, CA, USA) based on the local minima of normalized contact matrix [78,79]. To visualize the relationship between broad domains and TADs, we used HiCPlotter (version 0.7.3, Houston, TX, USA) [80].

### 4.10. Feature Enrichment Analysis within TAD Boundary Regions

To determine enrichment of broad domains within +/− 20 kb of the Boundary regions, we iteratively determined the expected distribution either CTCF, broad H3K4me3 or super-enhancer regions within our TAD boundary domains within 8354 randomly selected bins and iteratively repeated this 1000 times. We then calculated our observed estimation of the given regions (either for CTCF, broad H3K4me3 or super-enhancers) within the TAD Boundaries.

## Supplementary Material

Supplementary File 1
