# Supplementary material for: Disruption of Broad Epigenetic Domains in PDAC Cells by HAT Inhibitors
Source: Epigenomes. 2019 Jun 2;3(2):11. doi: 10.3390/epigenomes3020011 (PMC6897394; doi:10.3390/epigenomes3020011)
Supplement: Supplementary file 1 [file epigenomes-03-00011-s001.zip › epigenomes-499797-supplementary-final/Supplemental_Figure_Legends.pdf]

Supplemental Figure 1. Identification of super-enhancers in PANC1 cells. A. Super-enhancers were identified using Ranked Ordering of Super-Enhancers (ROSE) [5,65], where H3K27ac signal relative to input is ranked and visualized. The dashed line distinguishes between typical-enhancers and super-enhancers (Top). Genome snapshots of representative gene regions marked by super-enhancers in PANC1 (Bottom). **B.** We visualized the signal of core histone modifications including: H3K27ac, H3K4me1 and H3K4me3 within typical- or super-enhancers relative to gene regions (proximal <5 kb and distal >5 kb from the TSS). The signal is represented as log2 fold enrichment over input signal. **C.** Gene ontology analysis for 'Biological Process' was performed for typical- and super-enhancers.

Supplemental Figure 2. Identifying Broad H3K4me3 domains in PANC1 cells. A. Broad H3K4me3 domains were determined by MACS2 with the –broad flag activated. We then ranked the size of the domains and visualized them. The intersection of the dashed lines represents the cutoff of the top 5% of H3K4me3 sites, which we use to distinguish broad H3K4me3 domains from typical H3K4me3 regions. **B.** We then investigated the signal of core histone modifications including: H3K27ac, H3K4me1 and H3K4me3 within typical- and broad- H3K4me3 regions proximal (<5 kb) or distal (>5 kb) to TSS. The signal is representative of log2 Fold Enrichment over input for the given histone modification and the start and end indicates the broad region. **C.** Representative genome snapshots of regions containing broad H3K4me3 marks.

Supplemental Figure 3. Pathway analysis of super-enhancers and broad H3K4me3 domains in different PDAC grade groups.

Supplemental Figure 4. Gene expression relative to broad domains. Heatmaps showing the relative expression levels of genes marked by **A.** super-enhancers, **B.** broad H3K4me3, or **C.** both broad domains across 7 human PDAC cell lines. The number of genes belonging to each domain type is indicated. HGU corresponds to High-Grade Unique and LGU corresponds to Low-Grade Unique.

Supplemental Figure 5. The impact of ICG-001 treatment on broad H3K4me3 domains. A. Differential analysis of H3K4me3 genome-wide enrichment in PANC1 cells treated with ICG-001. Significantly altered regions are indicated by the colored dots (FDR <0.1). **B.** Genome browser snapshots of altered H3K4me3 regions. **C.** Differential binding analysis of broad H3K4me3 regions. **D.** Venn diagrams showing overlap of super-enhancers and broad H3K4me3 regions with increased histone modification levels.

Supplemental Figure 6. Chromatin-interacting domains in PANC1 cells. Genome-wide interaction matrix of chromosome contacts identified from TCC (left). Distribution of identified domain types using TopDom (40 kb resolution) (right).

Supplemental Figure 7. Domains with increased ChIP-seq signal after HAT inhibitor treatment are enriched in boundary regions. Feature enrichment analysis of super-enhancers (top) and broad H3K4me3 domains (bottom) increasing or decreasing in signal.
