# Supplementary material for: Disruption of Broad Epigenetic Domains in PDAC Cells by HAT Inhibitors
Source: Epigenomes. 2019 Jun 2;3(2):11. doi: 10.3390/epigenomes3020011 (PMC6897394; doi:10.3390/epigenomes3020011)
Supplement: Supplementary file 1 [file epigenomes-03-00011-s001.zip › epigenomes-499797-supplementary-final/Supplemental_Table S1.pdf]

**Sample**	**Replicate**	**Read count**	**% alignment**
PANC1 12hr ICG-001 H3K27ac	1	53,825,730	95.67
PANC1 12hr ICG-001 H3K27ac	2	47,544,286	93.88
PANC1 12hr ICG-001 H3K4me3	1	24,760,540	91.30
PANC1 12hr ICG-001 H3K4me3	2	46,725,697	98.00
PANC1 12hr C646 H3K27ac	1	45,877,530	96.56
PANC1 12hr C646 H3K27ac	2	45,578,483	96.32

Supplemental Table 1: Read count and percent alignment information for HAT inhibitors generated ChIP-seq datasets
