# Supplementary figures and images for: Disruption of Broad Epigenetic Domains in PDAC Cells by HAT Inhibitors
Source: Epigenomes. 2019 Jun 2;3(2):11. doi: 10.3390/epigenomes3020011 (PMC6897394; doi:10.3390/epigenomes3020011)

Supplemental Figure 1

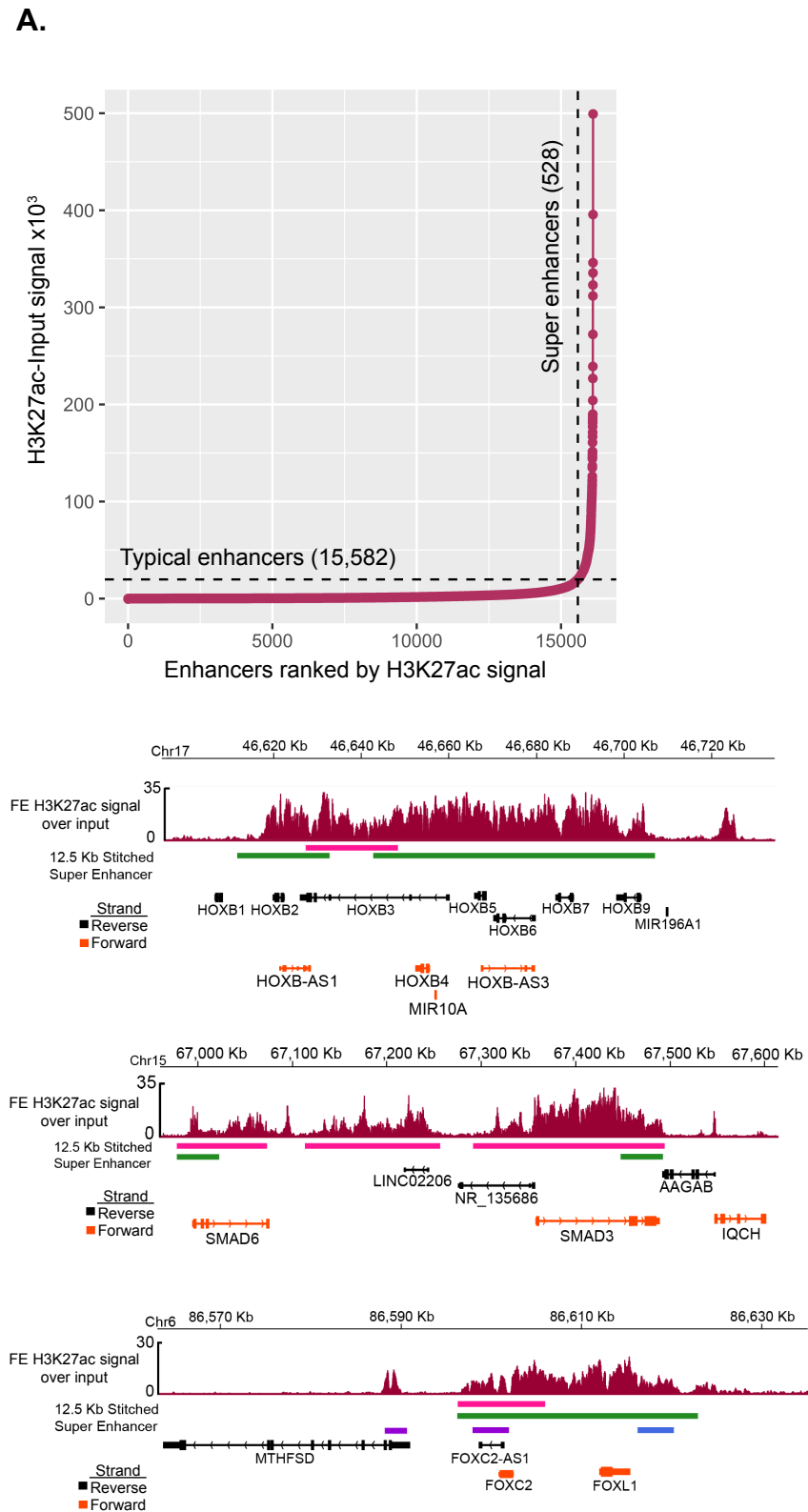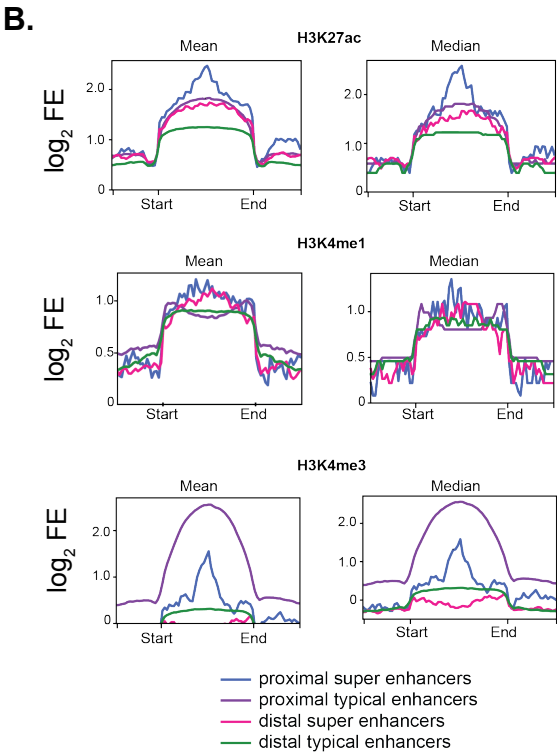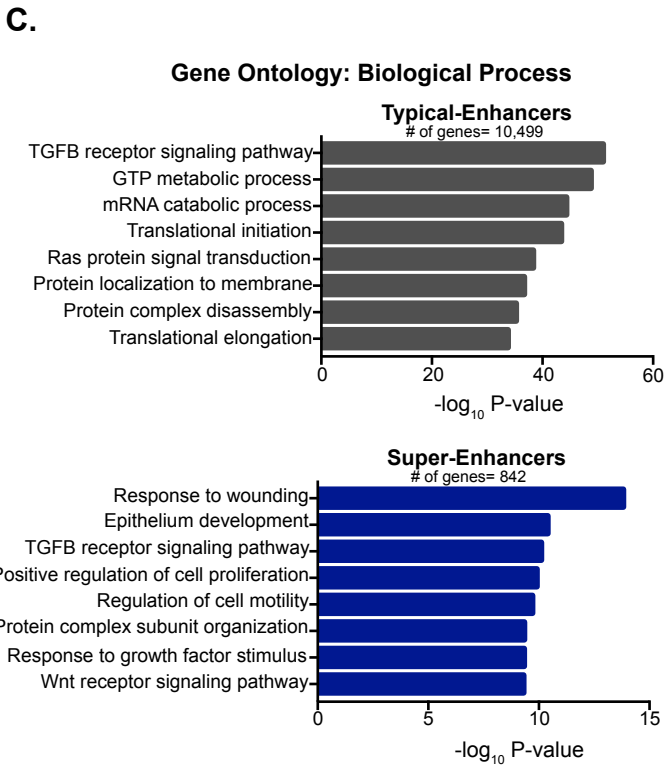

Supplement: Supplementary file 1 [file epigenomes-03-00011-s001.zip › epigenomes-499797-supplementary-final/Supplemental_Figure S1.pdf]

# Supplemental Figure 2

**A.**

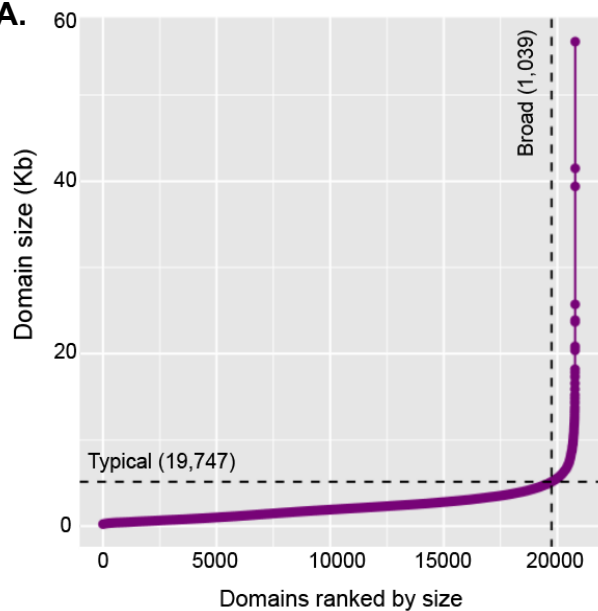

**B.**

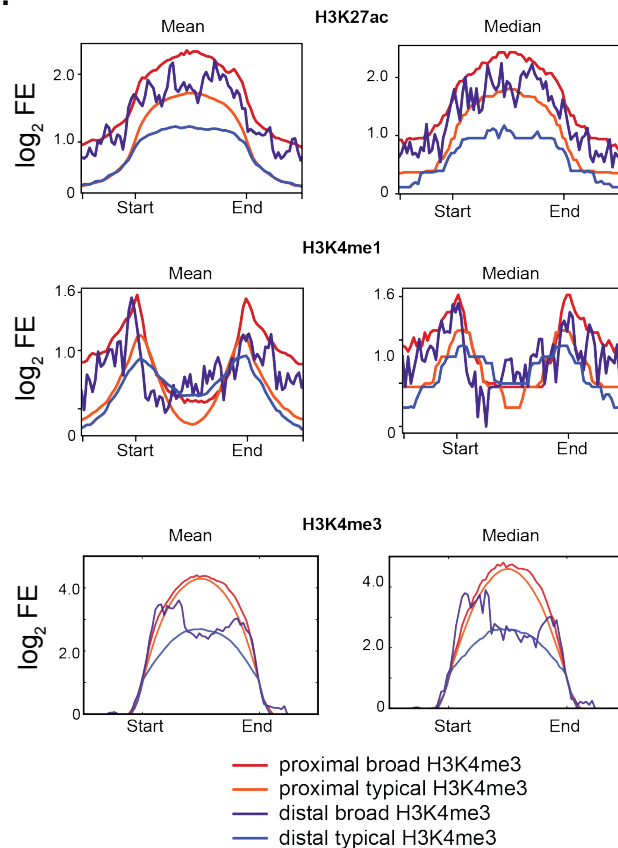

**C.**

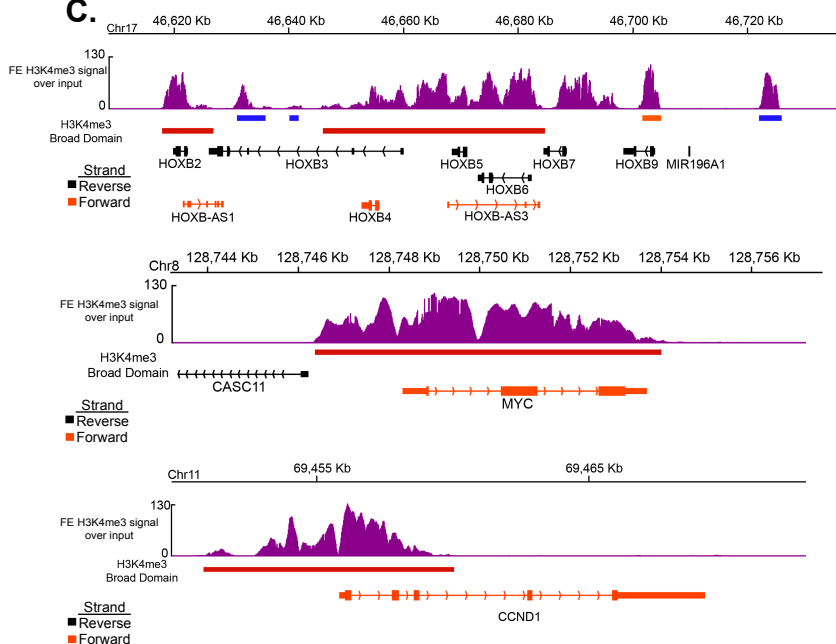

Supplement: Supplementary file 1 [file epigenomes-03-00011-s001.zip › epigenomes-499797-supplementary-final/Supplemental_Figure S2.pdf]

Supplemental Figure 3

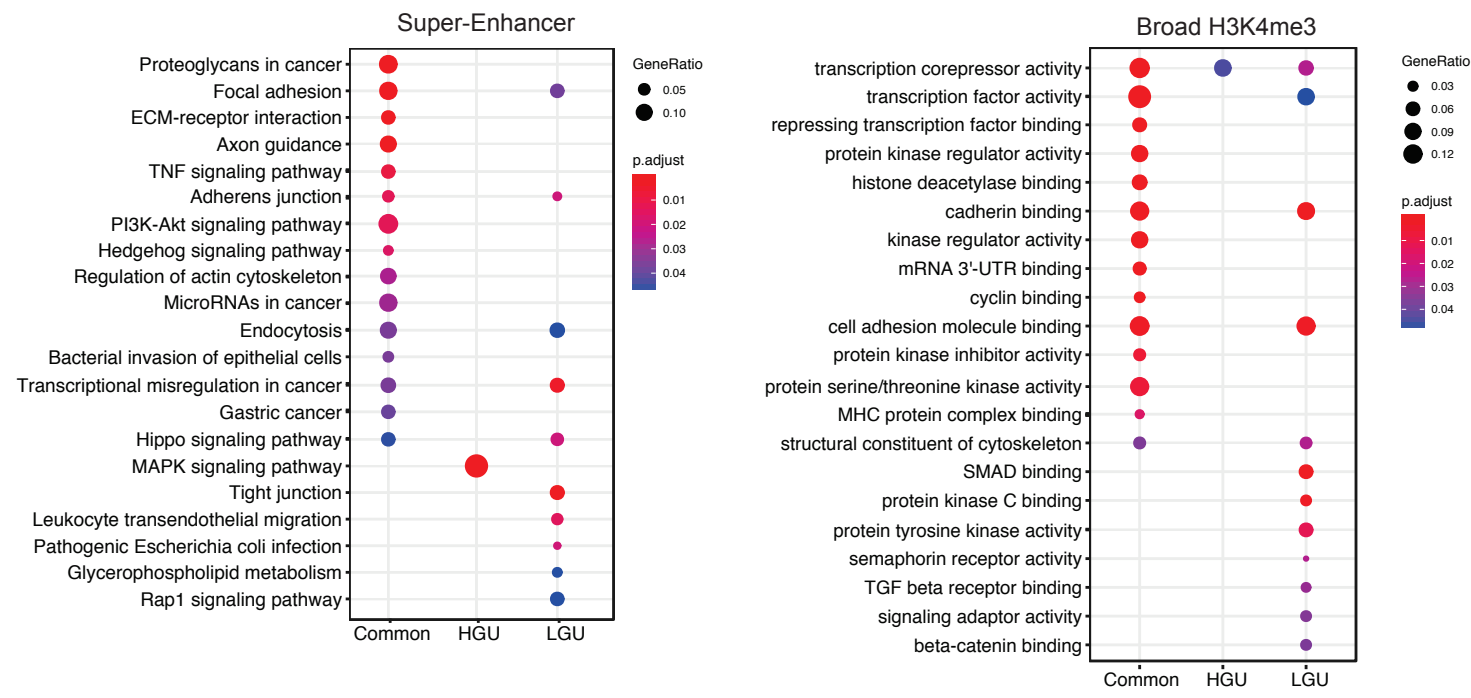

Supplement: Supplementary file 1 [file epigenomes-03-00011-s001.zip › epigenomes-499797-supplementary-final/Supplemental_Figure S3.pdf]

Supplemental Figure 4

A.

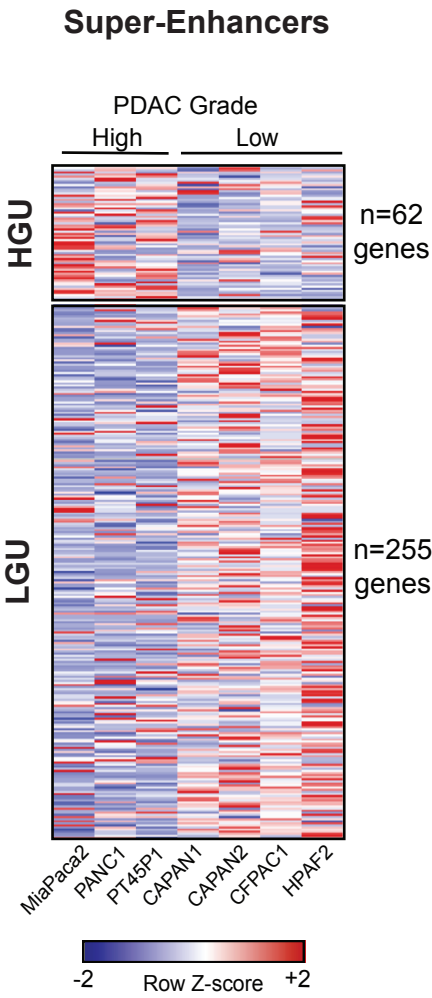

B.

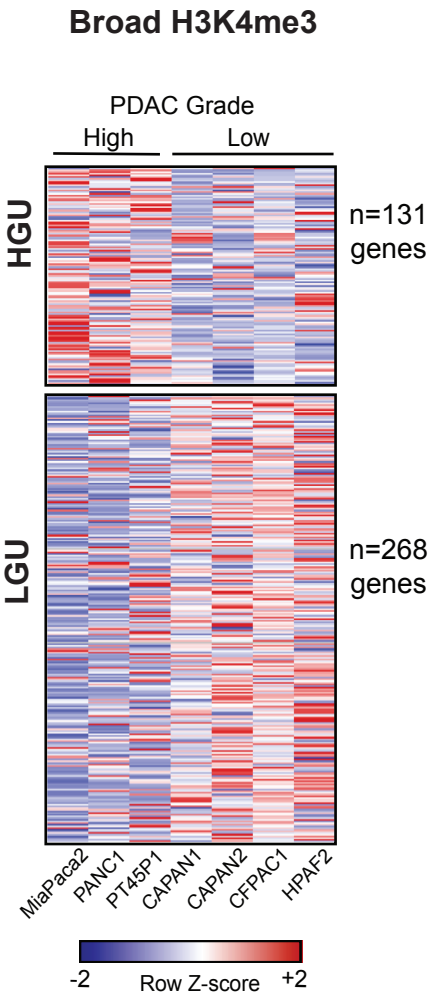

C.

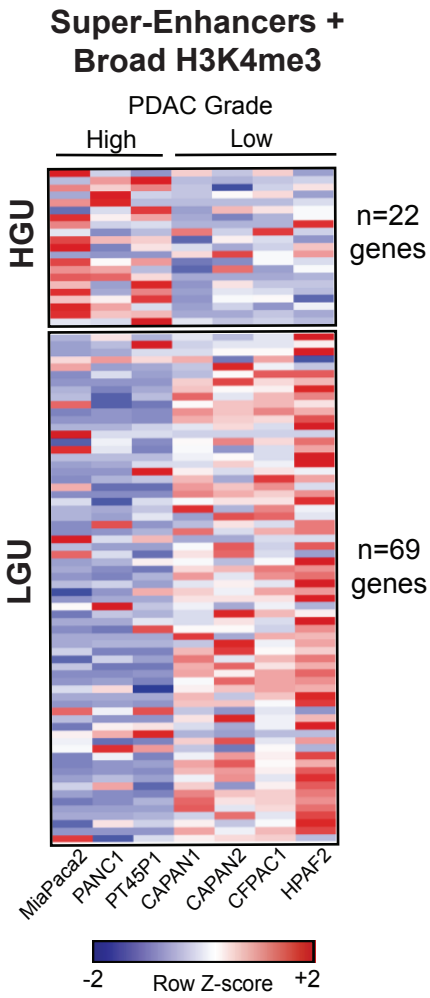

Supplement: Supplementary file 1 [file epigenomes-03-00011-s001.zip › epigenomes-499797-supplementary-final/Supplemental_Figure S4.pdf]

Supplemental Figure 5

A.

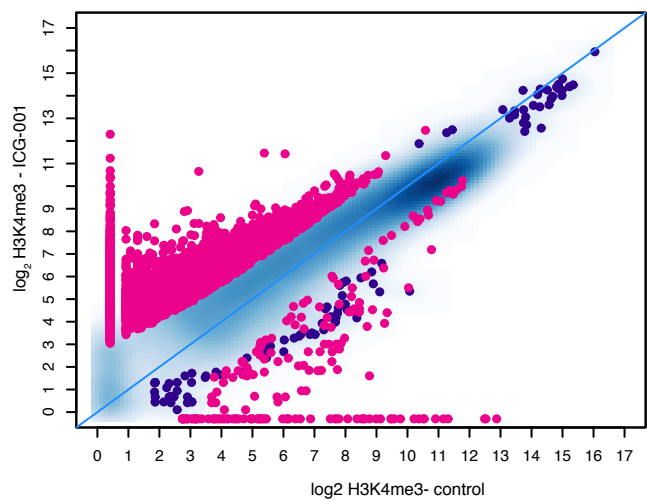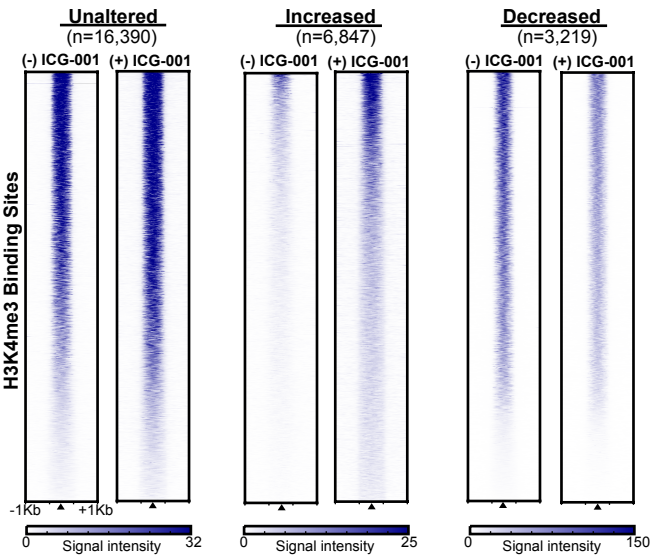

B.

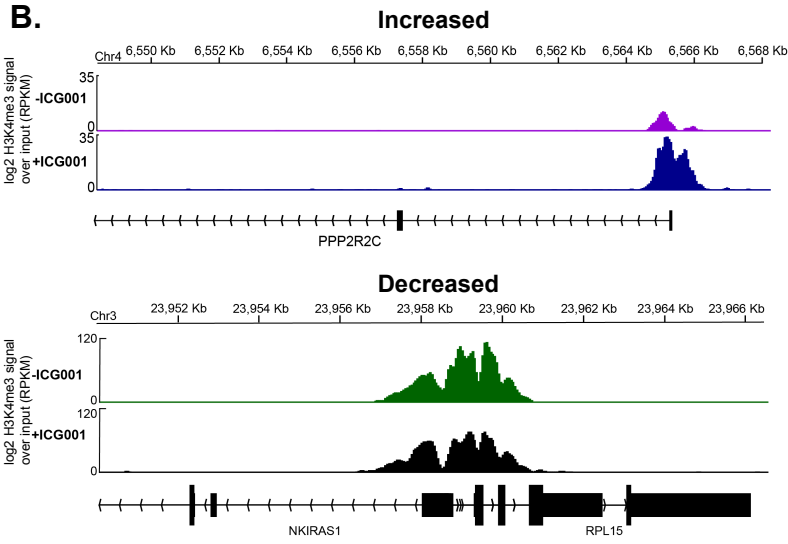

C.

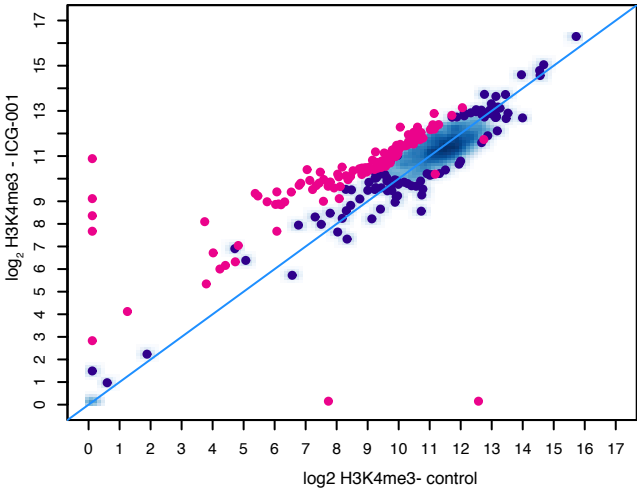

D.

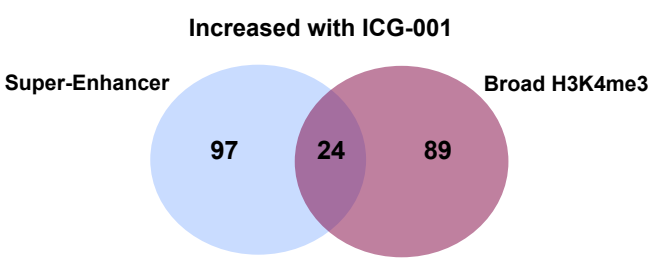

Supplement: Supplementary file 1 [file epigenomes-03-00011-s001.zip › epigenomes-499797-supplementary-final/Supplemental_Figure S5.pdf]

Supplemental Figure 6

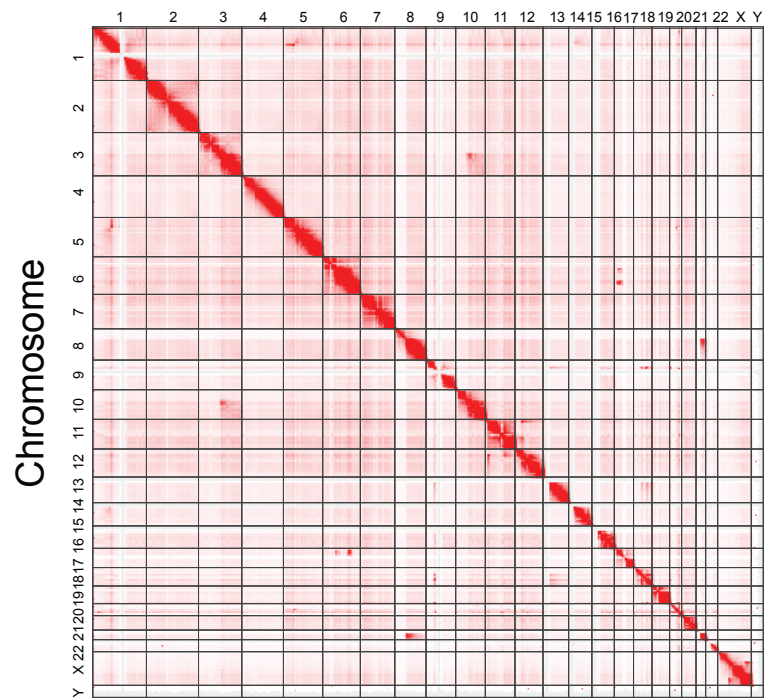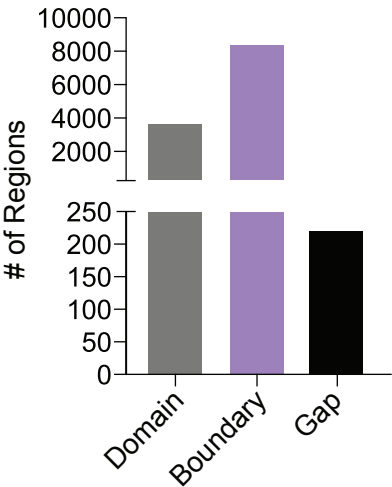

Supplement: Supplementary file 1 [file epigenomes-03-00011-s001.zip › epigenomes-499797-supplementary-final/Supplemental_Figure S6.pdf]

Supplemental Figure 7

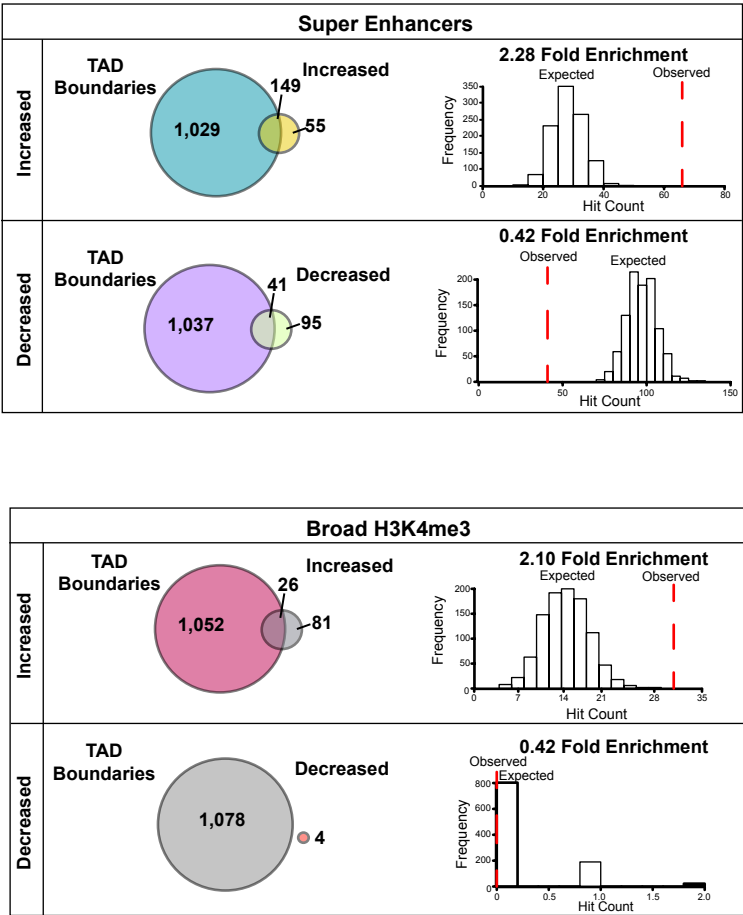

Supplement: Supplementary file 1 [file epigenomes-03-00011-s001.zip › epigenomes-499797-supplementary-final/Supplemental_Figure S7.pdf]
